# Annotations

**Published:** 1903-06-20

**Authors:** 


### Annotations.

### The Medical History of Mrs. Jane Welsh Carlyle.

Sir James Crichton-Browne, M.D., who is the Lord Chancellor's Visitor in Lunacy, has written an interesting introduction to a volume containing New Letters and Memorials of Jane Welsh Carlyle, the wife of Thomas Carlyle. He traces in his story the mental condition of Mrs. Carlyle, and shows that at the climacteric she passed through a mild but protracted attack of mental disturbance, which would be technically called on its psychical side climacteric melancholia, and on its physical side neurasthenia. Mrs. Carlyle, he says, was hereditarily predisposed to nervous disease. Her father, an able and vigorous man, died of typhus fever at 43 years of age, and there was a marked want of vitality in his family. He had twelve brothers and sisters, most of whom married; yet at the time of Mrs. Carlyle's death no heir was living in any branch to inherit the family house at Craigenputtock. Mrs. Carlyle's mother died of an apoplectic seizure, a maternal uncle was paralysed, and she derived her temperament from her mother's side of the family. Mrs. Welsh is said to have been in fifteen different humours in one evening, and was decidedly hot-tempered. Mrs. Carlyle's pathological tendencies had begun to develop themselves whilst she was still a girl, for there are complaints of sick headaches, and before her marriage these had got a firm hold of her. They were brought on by worry or excitement, even by the effort of talking, always by bodily vibration, as by travelling in a railway carriage, and were sometimes instantly arrested by a strong mental impression, their dependence on nerve storm being thus evinced. Besides the sick headaches she suffered from many, indeed innumerable, attacks of influenza. Harriet Martineau said she had "eight influenzas annually." And besides the influenza she had frequent catarrhs or colds, as she calls them, occurring almost invariably in spring and autumn. We know that such maladies depend to some extent on a special predisposition in the sufferer, having its root in the nervous system, upon which they leave their stamp in the way of undermining it. The instability and excitability of Mrs. Carlyle's nervous system is further shown by her intolerance of noise of all kinds as well as by her sleeplessness, which was even more than that of her husband. Within a year of her marriage in 1826 she writes, that she is "demolished" by a sleepless night; and in 1848 she wrote, "I sleep three hours a night, and that in small pieces." When the sleeplessness was combined with what she called one of her "patent" headaches, she sometimes passed into a state of unconsciousness. For several years before the climax of her illness she had been occasionally taking henbane, or hyoscyamus, and pretty frequently morphia. She was also addicted to excessive tea-drinking and smoked cigarettes. She began to complain of low spirits as early as 1841; but it was not until 1846, when she was 45 years of age, that her despondency assumed a morbid complexion. This deepened and darkened until 1855, and it was all but completely dispelled in 1857, leaving behind it, however, impaired bodily health and the seeds of serious evils in the nervous system, which afterwards sprouted and brought renewed depression of a very different nature from that previously experienced.

### A Lost Opportunity.

We have purposely refrained from referring to the blanket question until the material facts were known. The statement of Mr. Brodrick in the House of Commons shows the War Office to have once more broken down, for all the evils resulting from the sale are due to a monetary saving of some £1,500, or about 4d. a blanket. Further comment on this aspect of the case would be superfluous. It is, however, a pity that the policy, so to speak, of "shooting on sight" has been adopted in the case of the South African blankets, for thereby one question at least of scientific interest in connection with them will perforce remain unanswered. They are being "disinfected" or destroyed wherever found, without any attempt being made to distinguish simply dirty blankets from those specifically infected. It would have been easy enough to put aside for bacteriological examination a small percentage of each lot seized, and an estimate could then have been made of the total number infected. If this bore a large proportion to the whole, there would have been room for an inference, that the typhoid bacillus is capable not only of surviving for many months on damp textile fabrics under the circumstances of a voyage in a ship's hold, but of spreading from the site of its original deposition. It has been taken for granted of course, that the whole of the blankets were necessarily and originally infected, but for this there is hardly a vestige of foundation. A proportion of the blankets that accumulated and were eventually sold at Capetown were forwarded on from stations up-county, whither they had found their way after being lost or left behind near the lines of railway by the men. Among them there would naturally be some which belonged to men who were sick, and dropped them on their way to the hospitals from the veld and outlying camps. The great majority, however, either belonged to regiments which handed them in on their way home or were returned from the hospitals. Both the latter sources would be innocuous, for there is sufficient private and official evidence that the hospitals, sterilisers were in habitual use. However great, therefore, the number of dirty blankets, the proportion originally specifically infected must have been very small, but what the percentage was by the time they came to be distributed in England is another question. On this there is unfortunately no information. The blankets from the *Cornwall* are the only ones that have been adequately examined, and the evidence in regard to them was sufficiently strong, to make the inference that they were the cause of the outbreak, reasonable and natural. *Per contra* it has been shown that some of the blankets were used elsewhere without ill effect. Except indeed for the *Cornwall* case no specific ill has been traced to them, for though a case was reported from Bungay, the evidence was of the *post hoc, propter hoc* variety, and all possibility of real evidence removed by the blanket being destroyed. When all is said and done, perhaps the most remarkable point of the whole business is, that there should have been any sale at all for blankets so admittedly soiled and dirty as were these.

